# Aided and Unaided Decision-Making Among Partial Denture-Seeking Prosthodontic Patients Utilizing the Decisional Conflict Scale at the Dental Clinics of a College in Riyadh: A Questionnaire Study

**DOI:** 10.7759/cureus.77803

**Published:** 2025-01-22

**Authors:** Kiran Iyer, Reem Alghamdi, Mohammed Alsuhaibani, Abdullah Alyahya, Zaid Alonazi, Abdullah Alabdulwahab, Mozon Alshahrani

**Affiliations:** 1 Preventive Dental Sciences, College of Dentistry, King Saud Bin Abdulaziz University for Health Sciences, King Abdullah International Medical Research Center, Riyadh, SAU; 2 Dentistry, College of Dentistry, King Saud Bin Abdulaziz University for Health Sciences, Riyadh, SAU

**Keywords:** decision conflict scale, informed consent, patient’s satisfaction, removable and fixed prosthodontics, shared decision making

## Abstract

Background

The Decisional Conflict Scale (DCS) evaluates the decisional conflict patients experience during their healthcare-related decision-making process. There are various versions of the DCS, of which DCS-16 was originally recommended and put to use. It analyzes the patient treatment choice process on five subscales, hence, the study aimed to assess the influence of patient decision-making aid (PDA) on patients seeking prosthodontic care for partial dentures through the DCS.

Methods

All patients were randomly allocated into aided and unaided consultation groups, and patients seeking partial denture services were recruited from the college outpatient.

Results

The mean DCS score for the aided and unaided groups was 8 (±6.4) and 20.6 (±15.9), respectively. The age of the patient was significantly associated with the response to a question in the uncertainty sub-scale. In the aided category, the younger age group (20-40 years) was significantly (p = 0.00) less likely to feel sure about their decision on treatment options compared to the elderly age group (41-60 years) (OR: -17.8, CI: 1.47-2.28).

Conclusion

The present study revealed a significant association between decision-making among partial denture-seeking patients and their age, along with a substantial difference in decisional conflict between patients counseled using a standardized aid and unaided patients.

## Introduction

Dental professionals and patients have contrasting perspectives; the former focuses on technical aspects of dental treatment, and the latter emphasizes relief from the symptoms of oral disease. These differences stem from the value placed on dental treatment outcomes by the patient and the dentist. Dental treatment entails various options for a given condition, adding to the uncertainty of patient decisions [1].

Patient decision-making aids (PDAs) encourage shared decision-making and help patients make informed decisions about their health. They actively engage patients in decision-making by informing them of the pros and cons of various treatments available for the given condition. Certain evidence suggests that decision aids, including evidence-based information, are useful instruments for patient involvement, patient satisfaction, cooperation, and compliance. Scientifically validated PDAs must be adopted in numerous vernacular languages, for simplified, effective communication and cultural adaptation [2].

O'Connor put forward the definition of uncertainty regarding which course of action to take as a decisional conflict and was pivotal in developing the Decisional Conflict Scale (DCS) to evaluate the decisional conflict that patients experience during their healthcare-related decision-making process [3]. Prosthodontic services entail numerous treatment options ranging from removable partial dentures to fixed partial dentures for a missing dentition; the decision to opt among the treatment options depends on the value placed on functionality, aesthetics, cost, and the invasive nature of the procedure, to mention the least. Hence, decisional conflict arises when a person feels uninformed about treatment outcomes, is unsure how much personal values will be emphasized, and feels unaided in choosing or pressured to choose from one of the alternative treatments. Subjects with a high decisional conflict about the treatment options will usually delay making a decision. Ultimately, high decisional conflict compromises one's quality of life [3,4].

Studies from the past have reported that PDAs have been increasingly found and used in medicine, and are not emphasized much in dentistry, despite research evidence suggesting that there is a need to consider patient values and preferences when deciding on dental treatment [2,5]. Furthermore, there is evidence that dental patients prefer a collaborative decision-making style. A study by Johnson et al. used a decision aid for a preference-sensitive decision (treatment choices when root canal therapy or extraction is indicated) among patients, which yielded a small, but statistically significant improvement in patient knowledge of treatment options [6].

Very few studies have analyzed PDAs on the decision-conflict scale [3,6,7]. Hence, the aim of the present study was to develop the Arabic version of the DCS (16-item questionnaire) to assess the influence of PDA on patients seeking prosthodontic care for partial dentures at the dental hospital through the DCS. The PDA was developed in concurrence with the subject expert (American board-certified prosthodontist) for the study [5]. The objectives, towards this end, were to translate and validate the DCS in Arabic, develop a PDA based on evidence for partial dentures, analyze the role of exploratory variables in the decisional conflict between the groups (receiving consultation with and without aid), and to analyze satisfaction related to choice of treatment decision among the patients.

## Materials and methods

Study design and ethical considerations

A randomized control trial was conducted to assess the factors affecting decisional conflict score on the DCS-16 questionnaire among the patients seeking partial denture services at the Outpatient College of Dentistry, King Saud Bin Abdulaziz University for Health Sciences, Riyadh, Saudi Arabia. Before data collection, approval for the study was obtained from the Institutional Review Board (IRB) (IRB/0445/23) of King Abdullah International Medical Research Center (KAIMRC). Informed consent was obtained from all participants before being enrolled in the study. Patients unwilling to participate, and those unable to consent, were excluded from the study. The data collection for the study was carried out between May 24th, 2024, and November 2nd, 2024 (six months duration).

Sample size and sampling technique

The study adopted a probability-based simple random sampling technique. This method randomly allocated patients seeking partial denture services to the aided and unaided decision-making groups. The sample size was estimated at 26 samples in each group (aided and unaided) using G*Power software, version 3.1.9.4 (Heinrich-Heine-Universität Düsseldorf, Düsseldorf, Germany). 't'-tests (means, or differences between two independent means) were used for the estimation of sample size, with a confidence interval of 95%, a power of 80%, and an effect size of 0.50.

Translation of questionnaire

Data was collected using a questionnaire administered to patients seeking partial denture services by a blinded investigator during the clinical hours of the college. The DCS 16-item questionnaire has been used in various countries and validated in their respective vernacular languages. This study attempted to translate the English version of the DCS-16 item questionnaire into Arabic. A bilingual expert forward-translated the questionnaire into Arabic, and an independent translator then back-translated the same into English. Both translators were unaware of the concept studied, ensuring better validity [8]. Discussions were held between translators and investigators to arrive at a consensus. The investigators compared the back-translated questionnaire with the original English version to check for the integrity of the questions during the translation process. A pilot study was carried out on seven patients to assess test-retest reliability. The re-test was conducted among the pilot sample between these patients' first and second appointments. Internal consistency was assessed for reliability using Cronbach's alpha, item-total correlation, and item-delete statistics, which were calculated to check the internal consistency between the questions.

Questionnaire

The questionnaire consisted of two sections: demographic details and the DCS 16-item questionnaire. The demographic information gathered included name, gender, age, marital status, and monthly family income. Demographic variables were used as explanatory variables in the study. The DCS consists of five subscales: the informed subscale (three items), the values clarity subscale (three items), the support subscale (three items), the uncertainty subscale (three items), and the effective decision subscale (four items). Every item was scored on a five-point Likert scale. The sum of the scores for the DCS varies from 0 to 100 for every subscale. A score of 0 indicates the most favorable (positive) outcome, and 100 indicates the most negative outcome for the subscale. A high score would indicate a strong decisional conflict [3].

Aided and unaided decision-making process

Soon after the patient was screened at the College of Dentistry Outpatient (King Saud Bin Abdulaziz University for Health Sciences) and was indicated to receive partial denture service, the patient was allocated to the aided and unaided decision-making group based on the randomized method (the patients picked up concealed envelopes containing the group allocation and proceeded for consultation). The aided decision group comprised the PDA developed in concurrence with the subject expert, which the clinicians (senior undergraduate students) utilized to inform the patients about various partial denture treatment modalities, along with the evidence-based advantages and disadvantages. The unaided group received information/consultation from clinicians as a usual clinical process. The investigator administering the questionnaire was blinded to patient allocation.

Statistical analysis

The data was uploaded in Microsoft Excel (Microsoft® Corp., Redmond, WA, USA) and transferred to IBM SPSS Statistics for Windows, Version 20 (Released 2011; IBM Corp., Armonk, NY, USA) for analysis. Descriptive statistics were used to describe the demographic data based on frequency and percentage. Since the subscale scores are a continuous variable, the significance was evaluated between the two groups using the independent sample 't'-test. Multinomial analysis was carried out once the exploratory variables were found to be significant in Chi-square analysis.

## Results

A total of 76 patients were enrolled in the study, of which 38 were allocated randomly to receive aided or unaided consultation for the partial denture service. The mean age of participants was almost evenly distributed in both groups; the aided group had a mean age of 39.2 (±8.9), while the unaided group's mean age was observed to be 38.7 (±12). Both groups, when assessed, had a similar frequency and percentage distribution regarding marital status; in the aided group, 23 (60.5%) reported being married, whereas the unaided group consisted of 22 (57.8%) patients who reported being married. Gender distribution was kept equal in both groups during the enrollment period (Table 1). Similarly to age and marital status, interestingly, the number of patients in the wage bracket of SAR 5001-10000 was the highest in both groups. A high percentage of patients had visited the hospital for dental service in the aided (35, or 92.1%) and unaided (36, or 94.7%) groups, respectively. Post consultation, a high percentage of patients under the aided category opted for a fixed partial denture (27, or 71.1%). In contrast, regular consultation with the dentist for the unaided group observed a higher frequency and percentage of patients opting for the removable partial denture (25, or 65.8%) (Table 1).

**Table 1:** Demographic details of patients seeking partial denture service. SAR: Saudi Arabian Riyal

Demographic variable	Categories within variable		Total (N)	Mean	Standard deviation (±)	Frequency (n)	Percentage (%)
Age	Patients (aided)		38	39.2	8.9	-	-
	Patients (unaided)		38	38.7	12.0	-	-
Marital status	Patients (aided)	Single	38	-	-	11	28.9
		Married				23	60.5
		Separated				04	10.5
	Patients (unaided)	Single	38			15	39.4
		Married				22	57.8
		Separated				01	2.6
Family income (per month in SAR)	Patients (aided)	<5000	38	-	-	07	18.4
		5001-10000				15	39.4
		10001-15000				09	23.6
		15001-20000				05	13.1
		>20000				02	5.2
	Patients (unaided)	<5000	38	-	-	06	15.8
		5001-10000				18	47.4
		10001-15000				06	15.8
		15001-20000				06	15.8
		>20000				02	5.3
Have you visited the dentist before?	Patients (aided)	Yes	38	-	-	35	92.1
		No				03	7.9
	Patients (unaided)	Yes	38			36	94.7
		No				02	5.3
Which treatment option do you prefer?	Patients (aided)	Removable	38	-	-	09	23.7
		Fixed				27	71.1
		Implant				02	5.3
	Patients (unaided)	Removable	38	-	-	25	65.8
		Fixed				13	34.2
		Implant				00	-

The mean DCS-16 score was 6.3 (±4.4) for fixed partial denture-seeking patients who received aided consultation, compared to mean scores of 27.2 (±13.3) for fixed partial denture-seeking patients under the unaided consultation category. The aided category had a lower decisional conflict mean for fixed partial denture than for removable partial denture (Table 2).

**Table 2:** Partial denture preference of patients based on type of consultation (aided and unaided).

Type of consultation	Which treatment option do you prefer?	N	Mean	Std. deviation
Aided	Removable partial denture	9	13.4	8.8
	Fixed partial denture	27	6.3	4.4
Unaided	Removable partial denture	25	17.2	16.3
	Fixed partial denture	13	27.2	13.3

The independent sample ‘t’-test compared the DCS-16 mean scores of aided and unaided groups for their preference of removable partial denture and fixed partial denture; the aided group mean scores significantly (p = 0.00) reported a preference and less decisional conflict score for fixed partial denture, compared to the mean scores of the unaided group for fixed partial denture (Table 3).

**Table 3:** Independent sample ‘t’-test to analyze the significance of type of consultation on decision conflict scale scores. *p < 0.05 is considered as statistically significant

Type of consultation	t	df	Sig. (two-tailed)	Mean difference	95% confidence interval of the difference	
					Lower	Upper
Aided	3.17	34	0.00*	7.0	2.5	11.6
Unaided	-1.89	29	0.06	-9.9	-20.0	0.15

A negative correlation was predominantly observed for Q14 and Q15 under the effective decision subscale. Four other subscales (Informed Scale, Value Clarity Scale, Support Scale, and Uncertainty Subscale) of the DCS-16 questionnaire reflect high internal correlation and reliability (Table 4).

**Table 4:** Inter-item correlation matrix for Arabic translated DCS-16 scale. DCS: Decisional Conflict Scale

Inter-item correlation matrix																
	Q1	Q2	Q3	Q4	Q5	Q6	Q7	Q8	Q9	Q10	Q11	Q12	Q13	Q14	Q15	Q16
	Informed Scale			Value Clarity Scale			Support Scale			Uncertainty Subscale			Effective Decision Subscale			
Q1	1.00	0.581	0.486	0.493	0.263	0.306	0.105	0.199	0.125	0.307	0.361	0.286	0.120	-0.101	-0.005	0.136
Q2	0.581	1.00	0.426	0.314	0.105	0.310	0.102	0.237	0.245	0.315	0.217	0.290	0.116	0.209	0.180	0.241
Q3	0.486	0.426	1.00	0.601	0.287	0.673	0.487	0.393	-0.032	0.109	0.466	0.279	0.229	0.296	0.217	0.328
Q4	0.493	0.314	0.601	1.00	0.523	0.337	0.369	0.410	0.408	0.280	0.385	0.124	0.038	-0.009	0.459	0.263
Q5	0.263	0.105	0.287	0.523	1.00	0.337	0.064	0.213	0.374	0.421	0.468	0.232	0.022	-0.101	0.562	0.062
Q6	0.306	0.310	0.673	0.337	0.337	1.00	0.504	0.311	-0.101	0.091	0.409	0.250	0.239	0.091	-0.019	0.095
Q7	0.105	0.102	0.487	0.369	0.064	0.504	1.00	0.472	0.142	0.091	0.420	0.379	0.210	0.087	-0.001	0.082
Q8	0.199	0.237	0.393	0.410	0.213	0.311	0.472	1.00	0.077	0.025	0.404	0.182	0.384	0.183	-0.039	0.052
Q9	0.125	0.245	-0.032	0.408	0.374	-0.101	0.142	0.077	1.00	0.782	0.314	0.552	0.088	0.059	0.447	0.269
Q10	0.307	0.315	0.109	0.280	0.421	0.091	0.091	0.025	0.782	1.00	0.389	0.616	0.389	0.241	0.455	0.488
Q11	0.361	0.217	0.466	0.385	0.468	0.409	0.420	0.404	0.314	0.389	1.00	0.275	0.037	-0.172	0.055	-0.044
Q12	0.286	0.290	0.279	0.124	0.232	0.250	0.379	0.182	0.552	0.616	0.275	1.00	0.475	0.351	0.192	0.306
Q13	0.120	0.116	0.229	0.038	0.022	0.239	0.210	0.384	0.088	0.389	0.037	0.475	1.00	0.605	0.114	0.383
Q14	-0.101	0.209	0.296	-0.009	-0.101	0.091	0.087	0.183	0.059	0.241	-0.172	0.351	0.605	1.00	0.296	0.424
Q15	-0.005	0.180	0.217	0.459	0.562	-0.019	-0.001	-0.039	0.447	0.455	0.055	0.192	0.114	0.296	1.00	0.513
Q16	0.136	0.241	0.328	0.263	0.062	0.095	0.082	0.052	0.269	0.488	-0.044	0.306	0.383	0.424	0.513	1.00

The item-deleted statistics show consistent reliability for all 16 questions, meaning the removal of any question is unwarranted, and in the presence of all 16 questions, the reliability consistently remains the same, with a Cronbach’s alpha value of 0.84. The minimum reliability (0.82) value was observed for Q3: “Do you know the risks and side effects of each option?” and the maximum reliability (0.84) was observed for Q14: “Does your decision show what is important to you?” (Table 5).

**Table 5:** Item delete statistics to assess the influence of each question on the reliability of the scale.

DCS-16 item scale questions	Scale mean if item deleted	Cronbach's alpha if item deleted
Do you know which options are available to you?	7.18	0.833
Do you know the benefits of each option?	7.05	0.833
Do you know the risks and side effects of each option?	7.11	0.824
Are you clear about which benefits matter most to you?	7.16	0.826
Are you clear about which risks and side effects matter most to you?	7.03	0.832
Are you clear about which is more important to you (the benefits or the risks and side effects)?	7.05	0.833
Do you have enough support from others to make a choice?	7.08	0.833
Are you choosing without pressure from others?	7.00	0.834
Do you have enough advice to make a choice?	6.97	0.832
Are you clear about the best choice for you?	7.05	0.824
Do you feel sure about what to choose?	7.08	0.830
Is this decision easy for you to make?	6.37	0.832
Do you feel you have made an informed choice?	6.87	0.835
Does your decision show what is important to you?	7.16	0.841
Do you expect to stick with your decision?	7.29	0.836
Are you satisfied with your decision?	7.45	0.839

A Chi-square analysis of patients’ responses to the decision conflict scale, with that of independent variables for both aided and unaided categories, was carried out. Age, marital status, and previous visits to the dentist were observed to influence the response to questions in the aided category significantly. Age-related responses varied considerably for the questions from the effective decision subscale, such as “Do you feel you have made an informed choice?” and “Does your decision show what is important to you?” with significance values of p = 0.00 and p = 0.01, respectively (Table 6). Similarly, a significant response to one question each from the informed subscale, “Do you know which options are available to you?,” value clarity subscale, “Are you clear about what risks and side effects matter most to you?,” and uncertainty subscale, “Do you feel sure about what to choose?” were found to be significantly (p < 0.05) influenced by the previous visit to the dentist (Table 6).

**Table 6:** Chi-square analysis of aided category patients for decision conflict scale response along with independent variables. *p < 0.05 is considered as statistically significant

Variable	Categories	Question (aided), n (%)				Total	df	Chi-square test (p-value)
		Do you feel you have made an informed choice?					2	Chi-square value: 8.29; p = 0.01*
Age		Yes	Probably yes	Probably no	Unsure			
	20-40	16 (42.1)	3 (7.9)	0 (0)	4 (10.5)	23 (60.5)		
	41-60	4 (10.5)	8 (21.1)	0 (0)	3 (17.9)	15 (39.5)		
	Total	20 (52.6)	11 (28.9)	0 (0)	7 (18.4)	38 (100)		
Age		Does your decision show what is important to you?					2	Chi-square value: 11.84; p = 0.00*
		Yes	Probably yes	Probably no	Unsure	Total		
	20-40	21 (55.3)	0 (0)	0 (0)	2 (5.3)	23 (60.5)		
	41-60	7 (18.4)	6 (15.8)	0 (0)	2 (5.3)	15 (39.5)		
	Total	28 (73.7)	6 (15.8)	0 (0)	4 (10.5)	38 (100)		
Have you visited the dentist before?		Do you know which options are available to you?					3	Chi-square value: 12.39; p = 0.00*
		Yes	Probably yes	Probably no	Unsure	Total		
	Yes	27 (71.1)	6 (15.8)	2 (5.3%)	0 (0)	35 (92.1)		
	No	2 (5.3)	0 (0)	0 (0)	1 (2.6)	3 (7.9)		
	Total	29 (76.3)	6 (15.8)	2 (5.3)	1 (2.6)	38 (100)		
Have you visited the dentist before?		Are you clear about what risks and side effects matter most to you?					3	Chi-square value: 12.15; p = 0.00*
		Yes	Probably yes	Probably no	Unsure	Total		
	Yes	22 (57.9)	12 (31.6)	0 (0.6)	1 (2.6)	35 (92.1)		
	No	1 (2.6)	1 (2.6)	1 (2.6)	0 (0)	3 (7.9)		
	Total	23 (60.5)	13 (34.2)	1 (2.6)	1 (2.6)	38 (100)		
Have you visited the dentist before?		Do you feel sure about what to choose?					3	Chi-square value: 8.20; p = 0.04*
		Yes	Probably yes	Probably no	Unsure	Total		
	Yes	26 (68.4)	6 (15.8)	2 (5.3)	1 (2.6)	35 (92.1)		
	No	0 (0)	2 (5.3)	1 (2.6)	0 (0)	3 (7.9)		
	Total	29 (68.4)	8 (21.1)	3 (7.9)	1 (2.6)	38 (100)		

Independent variables, such as age, marital status, and previous visits to the dentist, significantly influenced responses to DCS questions from patients who received consultations under the unaided category. Questions such as “Are you clear about which is more important to you (the benefits or the risks and side effects)?” from the value clarity subscale varied significantly (p = 0.02) with age. The other two questions from the uncertainty subscale, namely “Are you clear about the best choice for you?” and “Do you feel sure about what to choose?” significantly varied in response based on marital status (p = 0.03) and a previous visit to the dentist (p = 0.00), respectively (Table 7).

**Table 7:** Chi-square analysis of decision conflict scale response for unaided category patients with independent variables. *p < 0.05 is considered as statistically significant

Variable	Categories	Question (unaided), n (%)					Total	df	Chi-square test (p-value)
		Are you clear about which is more important to you (the benefits or the risks and side effects)?						4	Chi-square value: 11.37; p = 0.02*
Age		Yes	Probably yes	Probably no	Unsure	No			
	20-40	5 (13.2)	3 (7.9)	1 (0)	10 (26.3)	4 (10.5)	23 (60.5)		
	41-60	8 (21.1)	2 (5.3)	3 (0)	0 (0)	2 (5.3)	15 (39.5)		
	Total	13 (34.2)	5 (13.2)	4 (30.5)	10 (26.3)	6 (15.8)	38 (100)		
Marital status		Are you clear about the best choice for you?						12	Chi-square value: 22.23; p = 0.03*
		Yes	Probably yes	Probably no	Unsure	No	Total		
	Single	0 (0)	1 (2.6)	5 (13.2)	2 (5.3)	3 (7.9)	11 (28.9)		
	Married	11 (28.9)	1 (2.6)	5 (13.2)	4 (10.5)	2 (5.3)	23 (60.5)		
	Separated/ widow(er)	2 (5.3)	2 (5.3)	0 (0)	0 (0)	0 (0)	4 (10.5)		
	Total	13 (34.2)	4 (10.5)	10 (26.3)	6 (15.8)	5 (13.2)	38 (100)		
Have you visited the dentist before?		Do you feel sure about what to choose?						5	Chi-square value: 13.27; p = 0.02*
		Yes	Probably yes	Probably no	Unsure	No	Total		
	Yes	14 (36.8)	3 (7.9)	10 (26.3)	4 (10.5)	4 (10.5)	35 (92.1)		
	No	0 (0)	2 (5.3)	0 (0)	0 (0)	1 (2.6)	3 (7.9)		
	Total	14 (36.8)	5 (13.2)	10 (26.3)	4 (10.5)	5 (13.2)	38 (100)		

The multinomial regression analysis between the question in the uncertainty subscale (“Do you feel sure about what to choose?”) and age, based on the type of consultation (aided and unaided), revealed a significant relationship. In the aided group, the younger age group (20-40 years) was significantly (p = 0.00) less likely to feel sure about their decision on treatment options compared to the elderly age group (41-60 years) (OR: -17.8, CI: 1.47-2.28). Whereas, in the unaided category, the elderly age group was significantly (p = 0.00) more sure of their choice compared to the younger age group (20-40 years) (OR: 18.68, CI: 13.38-34.94) (Table 8).

**Table 8:** Multinominal regression analysis of independent variable age with that of dependent variable confidence of treatment choice in patients. The last category was considered the reference group: No ^b^Reference Category among the age of patients; *p < 0.05 is considered as statistically significant

Variable		Independent variable	B	df	Sig.	Exp(B)	95% confidence interval for Exp(B)	
							Lower bound	Upper bound
Do you feel sure about what to choose? (aided)	Yes	Age (20-40)	-17.8	1	0.00	1.81	1.47	2.28
		Age (41-60)	0^b^	-	-	-	-	-
	Probably yes	Age (20-40)	-16.8	1	0.00	4.71	2.63	8.44
		Age (41-60)	0^b^	-	-	-	-	-
	Unsure	Age (20-40)	-17.2	1	0.00	3.14	3.41	4.62
		Age (41-60)	0^b^	-	-	-	-	-
Do you feel sure about what to choose? (unaided)	Yes	Age (20-40)	-0.99	1	0.05	0.37	0.04	3.01
		Age (41-60)	0^b^	-	-	-	-	-
	Probably yes	Age (20-40)	0.98	1	0.49	2.66	0.15	45.14
		Age (41-60)	0^b^	-	-	-	-	-
	Unsure	Age (20-40)	0.40	1	0.69	1.55	0.16	14.65
		Age (41-60)	0^b^	-	-	-	-	-
	Probably no	Age (20-40)	18.68	1	0.00*	13.03	13.38	34.94
		Age (41-60)	0^b^	-	-	-	-	-

## Discussion

Over the years, the DCS has been known for its reliability, sensitivity, and precise demarcation between various support systems that enable effective decision-making [9]. The scale was first developed in 1995. It attempts to detail the psychometric basis for patients who feel not appropriately informed or under-informed and are unclear about values and support systems for effective decision-making. The DCS has been translated and used in various vernacular languages to assess the decision-making process for multiple treatments in medicine and dentistry, where patients are in a profound dilemma of opting for treatment out of numerous options with known pros and cons as conveyed by clinicians [10].

The DCS-16 questionnaire has three formats (statement format, question format, and low literacy format); apart from these regular formats, one more version has been developed for clinicians (SURE test). In the present study, the "question format" was adopted and subjected to translation. The format consists of 16 questions with a five-point Likert scale response. Similar translations into vernacular languages have been carried out in various parts of the world; it has been adopted in 20 countries across five continents. Our study is the first to aim to translate the questionnaire into Arabic in the Middle East region (Gulf Cooperation Council) and apply it for assessment within the regional population [10,11].

A review on DCS states that the 16-item questionnaire has undergone less testing than its statement format DCS format [7]; the most common application of DCS has been in oncology and primary care [12]. Very few studies have attempted to apply the same in dentistry [13]. Dental treatment decision-making is complex and requires shared decisions from the clinician and the patient. Dental treatment considers meticulous rehabilitation of function and focuses on esthetical appeal. Loss of teeth impacts patients psychologically and socially and requires a considerable understanding of the treatment options available for decision-making. Replacement of lost teeth can be done through one of the two available alternatives: removable partial dentures, fixed partial dentures, or implants [14].

The present study attempted to understand the complexities involved for the patient while deciding on partial dentures and whether aided consultation could help the decision-making process. A literature search by the present study revealed only one study by Shrirao et al. that assessed patient decision-making for alternative treatments in partial dentures; the authors in the study did not use the DCS questionnaire for the same, but they elicited patient responses on a self-constructed questionnaire [14].

Decision support interventions (DESIs) use aids to support decision-making in comparison to usual care; the questionnaire tool has been used to test before or after making decisions [15,16]. In the present study, we used a visual aid with the contents of removable and fixed partial dentures; the aid was developed by board-certified senior faculty of prosthodontics, along with their advantages and disadvantages. The study is the first attempt in prosthodontics using the DCS questionnaire, which was previously only used in orthodontic research by Marshman et al., among parents and children undergoing fixed orthodontic appliance treatment. The DESIs used in the study by Marshman et al. were developed by a 10-member board in consultation with parents [2].

The mean DCS score of the unaided category in the study was similar to that of Kates [17], who evaluated the scale on cancer treatment. The average of all Cronbach’s alphas among the various versions of DCS has ranged between 0.71 and 0.88 [18-20]. For the traditional scale, Cronbach’s alpha has been elicited at >0.90 (0.70-0.97) in translated studies, except for a study by Lam et al., who translated the questionnaire into Mandarin Chinese [20]. The Cronbach’s alpha value, as observed in the study for Arabic translation, concurs with the above studies. In the present study, the internal consistency of the Arabic-translated questionnaire was perceived to be high, with all questions in the five subscales reflecting a Cronbach’s alpha of >0.83.

Our study observed that the choice of treatment was influenced by age; in the aided category, those in the age bracket of 41-60 years were more likely to feel secure with their decision using the visual aid for partial denture options than the younger age group of 20-40 years. In contrast, in the unaided group, those in the age group of 41-60 years were less likely to feel secure about their selection of partial denture options. This variable has not been compared with any of the studies, as it is being analyzed on the DCS scale for the first time.

The response to the DCS is known to vary between appointments, before and after treatment, and post-treatment follow-up. On similar lines, in the present study, both the aided and unaided groups seemed to be more confident/sure of their choice of treatment if they reported having previously visited the dentist. The reporting of confidence in the choice of treatment by patients with a previous dental visit is in line with a study by Sbaraini et al. [21].

There are several limitations to mention from the present study, such as factor analysis not being considered to assess the underlying psychometric analysis of the questionnaire. The patients in the study were recruited from a single public hospital that serves a particular population in the region and, hence, has a similar socio-economic status, which may be the reason for no discrepancy in the choice of treatment or influence of aid in decision-making. There is a need to recruit patients from diverse populations. Marital status does influence the choice of treatment and elicits replies on a support scale, as evident in the study; however, regression analysis of this variable did not provide significant results and has the potential to be explored further. DCS should be used in various clinical specialties of dentistry, as these specialties entail varying treatment options for a given condition. Clinicians should eventually use DCS data sets to train LLMs (Large Learning Models) so that AI (Artificial Intelligence) could prompt the patient to opt for appropriate treatment in the future.

## Conclusions

Various medical fields have utilized the DCS, with variations, for a couple of decades to understand the decision-making process among patients for treatment procedures with multiple options; dentistry has applied it sparingly. There is a need to encourage shared decisions and decisional conflict assessment for dental treatment preferences among patients. There is a need to encourage shared decision-making and create awareness among clinicians about decisional conflict assessment to acknowledge the patient's dental treatment preferences. The present study revealed a significant association between the variable age and decision-making for the type of partial denture based on aided and unaided consultation. Along with age, patients with a history of previous dental visits were more likely to be confident in their decision about treatment options.
